# AGE AND USE OF COMPLEMENTARY AND ALTERNATIVE MEDICINE

**DOI:** 10.1093/geroni/igz038.1842

**Published:** 2019-11-08

**Authors:** Sean N Halpin, Nivedita R Potapragaa, Sharon H Bergquist, Thomas Jarrett

**Affiliations:** 1 University of Georgia, Athens, Georgia, United States; 2 Emory University, Atlanta, Georgia, United States

## Abstract

Complementary and alternative medicines (CAM) are often obtained over the counter and not disclosed to health care practitioners—leading to possible unforeseen, harmful drug interactions. These concerns are especially true for older adults who have a high likelihood of experiencing multiple comorbidities. Yet few studies examine the patterns of CAM use and disclosure across a wide age range. We used a mixed-methods in a study on patient attitudes toward CAM in a large primary care setting. Participants (n=279) ranged in age from 21-85 (mean=58), were mostly white (75%), and had a bachelor’s degree or higher (83%). Most rated their physical health as good or very good (90%) and had a score of zero on the Charlson Comorbidity Index (76%). Use and disclosure of twelve types of CAM were assessed across three modalities including ingestible (e.g., herbs), psychological/mind-body (e.g., meditation), and physical (e.g., acupuncture). Age was not predictive of disclosure across the larger sample, but within respondents aged 65-85 (n=90), linear regression analyses showed likelihood of disclosure was associated with younger age, positive attitudes toward CAM, and expectation that their physician had positive attitudes about CAM. Semi-structured phone interviews (n=32) revealed older adults were more likely to have long-term CAM use, particularly for pain, and not feel it necessary to disclose to their physician. Meanwhile younger individuals reported trying CAM episodically for preventative health purposes. Understanding patterns of CAM use can help guide age-appropriate conversations and limit possible adverse outcomes from non-disclosure.

